# Three Self-Adhesive Resin Cements and Their Influence on the Marginal Adaptation of Zirconia-Reinforced Lithium Silicate Single Crowns: An In Vitro Scanning Electron Microscope Evaluation

**DOI:** 10.3390/jcm13113330

**Published:** 2024-06-05

**Authors:** Asaf Shely, Joseph Nissan, Diva Lugassy, Ofir Rosner, Eran Zenziper, Tharaa Egbaria, Gil Ben-Izhack

**Affiliations:** 1 Department of Oral Rehabilitation, The Maurice and Gabriela Goldschleger School of Dental Medicine, Sackler Faculty of Medicine, Tel Aviv University, Tel Aviv 6997801, Israel; asafshely@gmail.com (A.S.); nissandr@gmail.com (J.N.); rosnerop@yahoo.com (O.R.); eranzen@gmail.com (E.Z.); tharaae@mail.tau.ac.il (T.E.); 2 Department of Orthodontics, The Maurice and Gabriela Goldschleger School of Dental Medicine, Sackler Faculty of Medicine, Tel Aviv University, Tel Aviv 6997801, Israel; diva.lugassy@gmail.com

**Keywords:** prosthodontics, digital dentistry, self-adhesive resin cement, intraoral scanning, ZLS, dental materials, marginal gap, marginal adaptation, SEM, CAD-CAM

## Abstract

**Background**: In everyday dentistry, monolithic single crowns can be cemented with self-adhesive resin cements. The aim of this in vitro study was to evaluate how the marginal adaptation of full monolithic zirconia-reinforced lithium silicate (ZLS) single crowns is influenced by three different self-adhesive resin cements. **Methods**: Forty-five typodont teeth fully prepared for full monolithic crowns were divided into three groups (fifteen each) for the use of three different self-adhesive resin cements. A fourth control group (Temp-bond) was created by taking five teeth from each group before cementation with self-adhesive resin cements. All forty-five abutments were scanned using a Primescan intra-oral scanner (IOS), followed by computer-aided design (CAD) and computer-aided manufacturing (CAM) of zirconia-reinforced lithium silicate (ZLS) full crowns using a four-axis machine. Initially, the crowns of the control group were fixed to the abutments using Temp-bond, and the marginal gap was evaluated using a scanning electron microscope (SEM). After removing the control group crowns from the abutments, fifteen crowns in each group were cemented using a different self-adhesive resin cement and observed under SEM for evaluation of the marginal gap. A Kolmogorov–Smirnov test was performed, indicating no normal distribution (*p* < 0.05), followed by Mann–Whitney tests (α = 0.05). **Results**: The total mean marginal gap of the temp-bond control group was significantly lower compared to all three groups of self-adhesive resin cement (*p* < 0.0005). The total mean marginal gap of the G-cem ONE group was significantly lower compared to the TheraCem group (*p* < 0.026) and RelyX U200 group (*p* < 0.008). The total mean marginal gap of the TheraCem group was significantly higher than the G-cem ONE group (*p* < 0.026) but showed no significant difference with the RelyX U200 group (*p* > 0.110). **Conclusions**: All four groups showed a clinically acceptable marginal gap (<120 microns). Although all three groups of self-adhesive resin cement showed a significant increase in the marginal gap compared to the temp-bond control group, they were within the limits of clinical acceptability. Regarding the marginal gap, in everyday dentistry, it is acceptable to use all three self-adhesive resin cements, although the G-cem ONE group exhibited the lowest marginal gap for ZLS single crowns.

## 1. Introduction

In prosthetic dentistry, interest in achieving nonmetallic and functionally biocompatible materials has increased [1]. The predictable strength and good longevity have made traditional metal-ceramic restorations popular. On the other hand, patients’ demand for improved esthetics has driven the development of metal-free dental ceramics [2]. Many researchers and companies have tried to find new methods to reinforce all-ceramic restorations, with the main goal of maintaining a high-strength core and a more natural look, with an emphasis on an acceptable marginal gap. Today, the clinical use of all-ceramic crowns has extended to the complete arch due to their natural appearance and biocompatibility [3].

The high demand for restorations with increased translucency and good biocompatibility led to the introduction of zirconia-reinforced lithium silicate ceramics (ZLS). The ZLS, marketed as CELTRA^®^ DUO (CELTRA^®^ DUO, Sirona Dentsply, Milford, DE, USA), is a homogenous glassy matrix consisting of lithium meta-silicate and lithium ortho-phosphate grains combined with tetragonal zirconia fillers. This promising hybrid ceramic material has been found to have high biological and mechanical properties that can withstand physiological occlusal forces at a 1 mm thickness [4]. It also exhibits improved volumetric shrinkage due to the firing process and a clinically acceptable marginal gap (<150 µm) [5,6]. 

Marginal adaptation plays a crucial role in the long-term success of dental restorations. Inadequate marginal adaption is associated with increased plaque retention, cement dissolution, endodontic infection with pulp lesions, and periodontal diseases, which can lead to bone resorption [1,3] There has been controversy over the precise definition of “marginal adaption”. Holmes et al. stated that the fit of the casting is examined by measuring certain points between the casting surface and the tooth. He defined the term as the perpendicular measurement from the marginal surface of the casting to the axial wall of the preparation [7]. 

Cement is an essential factor in determining the success of the restorations [8]. Many studies have advocated that a marginal gap less than 120 µm is clinically acceptable, and the achievable range may vary depending on factors like the restoration material and finish line configuration [8,9,10,11]. This includes McLean et al., who also suggested that 120 µm is the maximum clinical discrepancy that can be tolerated [10]. Some investigations attribute this conclusion to the decrease in facture resistance [11]. On the other hand, Contrepois et. al reviewed many studies pertaining to the marginal fit of ceramic crowns fabricated with different systems and found that it clinically ranges from 3.7 to 174 µm [3].

It has been shown that crowns with reduced cement spacers lead to an increased marginal discrepancy due to a reduction in the passive fit on the abutment. A study that evaluated the marginal gap adaption of CELTRA^®^ DUO (CELTRA^®^ DUO, Sirona Dentsply, Milford, DE, USA) with different digital radial spacers set at 60, 90, and 120 µm and measured the marginal gap in four regions by scanning electron microscope (SEM) concluded that the preferable radial spacer for CELTRA^®^ DUO (CELTRA^®^ DUO, Sirona Dentsply, Milford, DE, USA) crowns is 90 µm [12].

Luting cements are classified into two main categories: water-based and resin-based polymerizing cements [13,14]. Water-based cements such as glass ionomer and zinc phosphate cements have been wildly used throughout history due to their conventionality.

However, resin-based cements form a strong mechanical bond and have low solubility. In addition, some may also form a chemical bond with their substances. They have better physical properties and demonstrate better retention compared with water-based cements [15]. Due to their advanced features, many researchers, including Ganapathy et al. and others, have argued that the use of resin cements is preferred in all-ceramic restorations [16,17,18]. 

Surface pretreatment is a pivotal technique in resin-based cements. It includes etching with hydroxyl-apatite followed by salinization and applying a bonding agent. The backlash of this technique is that the pretreatment steps self-jeopardize the restoration’s bonding strength due to many devastating factors, such as a possible contamination [19].

Therefore, self-adhesive resins were developed in 2002 to overcome this backlash. They bond to dentin and enamel with no surface pretreatment required. The application of self-adhesive resin cements is accomplished with only a single step, eliminating the adhesive steps, making the process much more facilitated and simpler [19].

We know from the literature that self-adhesive resin cement is much more viscous compared conventional resin cement, which may affect the marginal gap. It also has a lower acidic monomer concentration compared to conventional resin cements, and infiltration into the tooth structure is limited [20].

As we increase the filler content in resin cement, we also increase the viscosity of the cement, and this also affects the bond strength of glass ceramic, depending on the surface treatment [21].

CELTRA^®^ DUO is a relatively new material in the industry, and there are new SARCS emerging into the market. It is very interesting to examine the effects of these new SARCS on the marginal gap in this material. This research is novel regarding the cements that we used and the types of material.

After a comprehensive search of the current literature, we were not able to find any studies that compared the influence of different self-adhesive resin cements on the marginal gap of zirconia-reinforced lithium disilicate (CELTRA^®^ DUO) single crowns. The null hypothesis was that no difference would be found between the three different self-adhesive resin cements.

## 2. Materials and Methods

**Figure 1:**
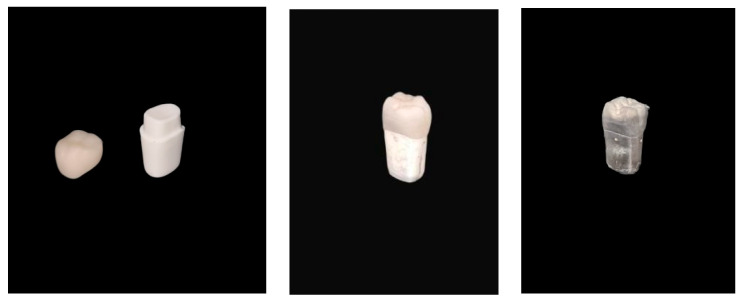
Plastic tooth of the first maxillary molar with crown (zirconia-reinforced lithium silicate) before and after gold coating.

Forty-five machine-made identical plastic teeth (FLUX 8634; Columbia Dentoform, Lancaster, PA, USA, upper right first molars) were used. All the teeth had an occlusal clearance of 2 mm, an axial convergence angle of 6 degrees, and a shoulder finish line configuration at the width of 1.2 mm all around (Figure 1). All forty-five abutments were scanned with an intra-oral scanner (IOS) (CEREC^®^ Primescan; Dentsply Sirona, Charlotte, NC, USA). A virtual model (CEREC^®^ SW 5.2.4; Dentsply Sirona) was created; a well-experienced single user (G.B.I., 9 years of experience) marked the finish line; and a virtual single-fix partial denture design (CEREC^®^ SW 5.2.4; Dentsply Sirona) was created with the following digital parameters: radial spacer: 90 µm, occlusal spacer: 120 µm, proximal contacts strength: 25 µm, occlusal contacts strength: 25 µm, radial minimal thickness: 1000 µm, occlusal minimal thickness: 1500 µm, margin thickness: 100 µm, margin ramp width: 100 µm, and margin ramp angle: 60°. It is important to mention that, due to the results of a previous study [12], we used a 90 µm radial spacer for all ZLS single crowns. The forty-five abutments were divided into three groups of fifteen each:

Group 1: G-cem ONE Automix (GCA; GC, Alsip, IL, USA).
Group 2: TheraCem Automix (BISCO, Inc. Schaumburg, IL, USA).
Group 3: RelyX U200 Automix (RXU200; 3M ESPE, Seefeld, Germany).

A four-axes chair-side milling machine (CEREC MC XL^®^; Dentsply Sirona) was used to produce forty-five single-fixed partial dentures from zirconia-reinforced lithium silicate (ZLS) (CELTRA^®^ DUO, Sirona Dentsply, Milford, DE, USA).

Before using the self-adhesive resin cement, five plastic teeth and crowns were taken from each of the three groups, and a fourth control group of fifteen units (crown and abutment) was created. The control group gives a baseline that we can compare to the results of the other three groups. The crowns were fixed to the abutments with temp-bond (Temp-Bond™ NE™ Unidose; KaVo Kerr, Brea, CA, USA), and it is important to mention that the use of a small amount of temp-bond on the intaglio of the crowns was solely for the fixation of the crowns on the abutments so that they would not move during measurements under SEM. After the following measurement protocol (which is described below), we removed the crowns, cleaned the temp-bond from the crowns and the abutments with a steamer, and made the cementation with the self-adhesive resin cement from each group.

Following the manufacturer’s recommendations, it was possible to use these self-adhesive resin cements without any preparation inside the crown or on the abutment. The cementations were performed by an experienced dentist (A.S.). All the cementation procedures were performed by auto-mixed cement to apply the cement with standardization. Each crown was half filled, and the cement was smeared at the borders of the crown. 

For each group during the cementation, constant pressure was applied. To achieve an achieving fit, it was performed using Lutron Electronic Enterprise Co., Ltd. FG-20 KG (Lutron Electronic Enterprise Co., Ltd., Taipei City, Taiwan), which was set to 50 N.cm. until the setting time was completed, as recommended by the manufacture.

**Figure 2:**
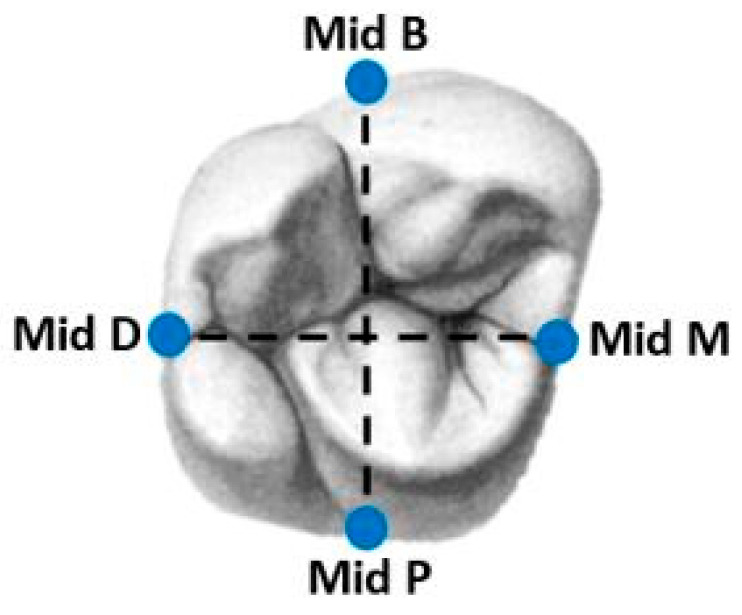
Four repeatable reference points in the middle (mid) of each side.

On each side of the plastic tooth, we marked a dot with a tungsten bur (FG330; Strauss&Co, Ra’anana, Israel) to receive four repeatable reference points for the measurements under SEM. These points were located in the mid-buccal, mid-mesial, mid-distal, and mid-lingual areas (Figure 2). In each unit, we used a mini sputter coater (SC7620; Quorum Technologies, Lewes, Britain) for 45 s, as this coating is necessary when using scanning electron microscope (SEM) (Figure 1).

**Figure 3:**
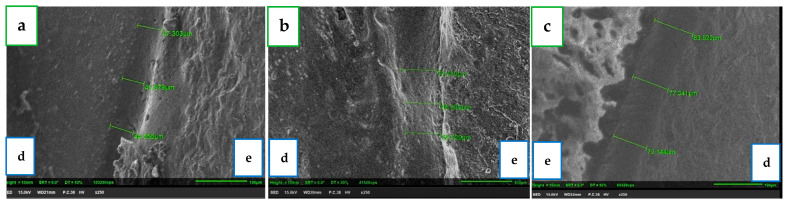
A side view of the three measurements at a reference point under SEM of all three self-adhesive resin cement groups. From left to right: (**a**) G-cem ONE; (**b**) TheraCem; (**c**) RelyX U200; Green line—marginal gap. d—Side of plastic tooth; e—Side of the ZLS crown. The average of every three me asurements is defined as CMG.

The marginal gap was evaluated by SEM (JSM-IT100; JEOL, Akishima, Tokyo, Japan) with a magnification of ×250 and measurement software (InTouchScope, https://www.jeol.co.jp/products/scientific/sem/JSM-IT500.html, accessed on 15 March 2024). All measurements were performed by a single experienced user (A.S.). Three measurements were taken at each one of the four sites (Figure 3) to calculate the average of these three measurements and define it as the circumferential marginal gap (CMG). The mean marginal gap (MMG) for each surface (distal, mesial, buccal, and palatal) was calculated as the average of the fifteen CMGs at each site for each of the four groups. For each group (15 units), we received a total of 180 measurements, and the total mean marginal gap (TMMG) was defined as the average of these measurements. In total, there were four groups (temp-bond and three self-adhesive resin cements) and 720 measurements (180 for each group). In each group, the MMG was calculated for each of the four surfaces (distal, mesial, buccal, and palatal) as the average of the fifteen CMG. The average of all 180 measurements in each group was defined as TMMG. 

The statistical analysis was performed using statistical analysis software, Statistical Package for Social Sciences for Windows Release 24.0 (SPSS Inc., Chicago, IL, USA). 

A Kolmogorov–Smirnov test was used on the study variables, which indicated no normal distribution (*p* < 0.05). A Mann–Whitney test was used for a comparison of MMG and TMMG between the four groups. The statistical significance level for this work is *p* < 0.05.

## 3. Results

A Kolmogorov–Smirnov test was performed on the study variables, which indicated no normal distribution (*p* < 0.05). A sensitivity power analysis was performed using G*power to calculate whether the size of the effect in our study was sensitive enough to be detected. A Mann–Whitney test with two groups, *n* = 15 and *n* = 15, would be sensitive to the effect of Cohen’s d = 1.08 with 80% power (α = 0.05, two tailed). All four groups were observed and measured in four regions of interest (Figure 3). The median and percentiles of the mean marginal gap (MMG) of each surface (distal, mesial, buccal, and palatal) (Table 1) and the median and percentiles of the total mean marginal gap (TMMG) of each cement group (Table 2) were calculated.

Accordingly, Mann–Whitney tests were used and revealed a significantly lower mean marginal gap (MMG) for all four surfaces (distal, mesial, palatal, and buccal) of the control group (temp-bond) compared to all four surfaces (distal, mesial, palatal, and buccal) of the three groups of the self-adhesive resin cement (G-cem ONE (*p* < 0.0005), TheraCem (*p* < 0.0005), and RelyX U200 (*p* < 0.0005)) (Table 1). The control group (temp-bond) also demonstrated a significantly lower total mean marginal gap (TMMG) in comparison to all three self-adhesive resin cement groups (G-cem ONE (*p* < 0.0005), TheraCem (*p* < 0.0005), and RelyX U200 (*p* < 0.0005)) (Table 2). 

The G-cem ONE group revealed a significantly lower mean marginal gap (MMG) for all four surfaces (distal, mesial, palatal, and buccal) and total mean marginal gap (TMMG) compared to the TheraCem group (*p* < 0.026) and RelyX U200 group (*p* < 0.008) (Table 1 and Table 2). 

The mean marginal gap (MMG) for all four surfaces (distal, mesial, palatal, and buccal) and the total mean marginal gap (TMMG) of the TheraCem group were significantly higher than for the G-cem ONE group (*p* < 0.026) but showed no significant difference to the RelyX U200 group (*p* > 0.110).

**Figure 4:**
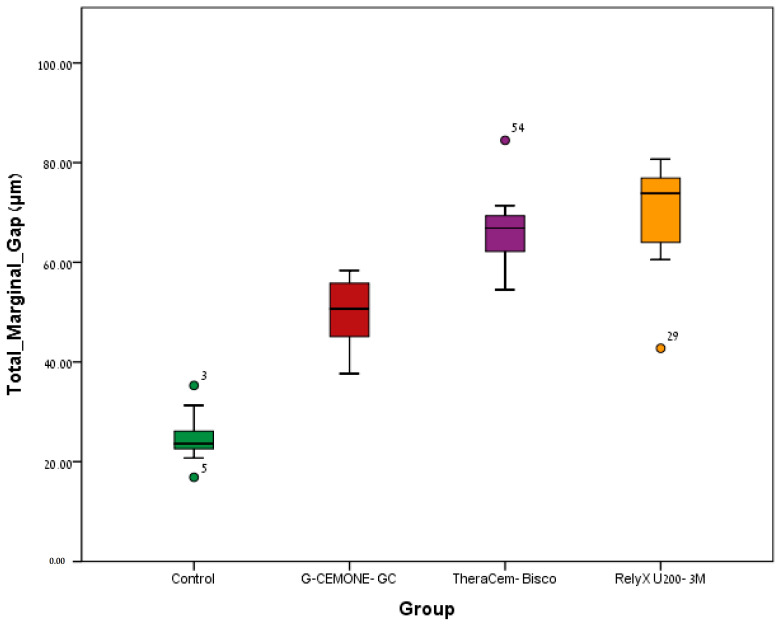
Median (P50), P25, and P75 of the total mean marginal gap (μm) of the four groups: control (temp-bond); G-cem ONE; TheraCem; and RelyX U200.

For the control group, the total mean marginal gap median was 23.60 μm; for the G-cem ONE group, the total mean marginal gap median was 50.66 μm; for the TheraCem group, the total mean marginal gap median was 66.87 μm; and for the RelyX U200 group, the total mean marginal gap median was 73.83 μm (Figure 4).

Due to the results of the statistical analysis, the null hypothesis must be rejected, as significant differences were found in the MMG and TMMG between the four groups (Figure 4). The control group (temp-bond) had significantly lower MMG and TMMG compared to all three groups of self-adhesive resin cement (*p* < 0.0005). The G-cem ONE group had significantly lower TMMG compared to the TheraCem group (*p* < 0.026) and RelyX U200 group (*p* < 0.008). There was no difference between the TheraCem group and the RelyX U200 group (*p* > 0.110).

## 4. Discussion

While seeking articles exploring different and relatively new self-adhesive resin cements and their influence on the marginal gap, no articles were found. The null hypothesis was rejected as there was a significant difference between the four groups. The control group (temp-bond) exhibited significantly lower MMG and TMMG compared to all of the other three groups of self-adhesive resin cement. The G-cem ONE group exhibited significantly lower MMG and TMMG compared to the TheraCem group and the RelyX U200 group. Between the last two groups (TheraCem and RelyX U200), no significant differences were found. 

Habib et al. examined zirconia crowns without cement and one group with resin cement (RelyX Unicem) without venting holes. In their study, the group without cement had a mean marginal gap of 126.11 μm, while the group with the resin had a mean marginal gap of 143.04 μm. The difference between the two groups was about 17 μm, while in our study, the lowest difference between the temp bond group and the G-cem ONE group was around 27 μm, which is close. The overall results in Habib’s study are much higher than the results in this study and exceed 120 μm. This can be explained by the research limitations: they used a different material; the specimens went through 1500 cycles of thermocycling; they used a different IOS with lower scanning technology; and they measured with a digital microscope, which is less accurate than SEM [22].

A study by Pilo et al. examined the cementation of monolithic zirconia with four groups of self-adhesive resin cements (RelyX U200, SmartCem 2, G-Cem Automix, and Panavia 21) and the influence it has on the marginal gap, before and after cementation. Their results are equivalent to the results in this study, as there was a significant difference before and after cementation. Before cementation, the mean marginal gap was 34.83 μm, and in our study, it was 23.60 μm. After cementation, the mean marginal gap was 72.00 μm compared to 50.66–73.83 μm in this study. In Pilo’s study, the differences before and after cementation were around 37 μm, while in this study, the differences were between 27 and 50 μm. These differences are quite similar [23].

A recent study by Shely et al. examined the effect of post-crystallization of ZLS single crowns on the marginal gap. Plastic teeth were used and scanned by an intraoral scanner (Primescan), and CELTRA^®^ DUO single crowns were grinned by a four-axis milling machine and cemented with temp-bond. Before crystallization, the mean marginal gap was 24 μm, and after crystallization, the mean marginal gap increased to 80 μm. In this study, the same methods were used but without the post-crystallization process. For the temp-bond group, TMMG was 24 μm, and TMMG was 50–73 μm for self-adhesive resin cements. In the previous study, crystallization increased the marginal gap of CELTRA^®^ DUO by 56 μm, and in this study, the self-adhesive resin cements increased the marginal gap between 27 and 50 μm. When combining these two results, it can be expected that post-crystallized CELTRA^®^ DUO single crowns with self-adhesive resin cement may have a gap of around 106–126 μm. Such a gap is around the clinically accepted threshold, and it is known that the gap may increase even more when working in an in vivo environment, which has more limitations [24]. 

Finish line configuration is a very important factor that may affect the marginal gap adaptation. It is one of the most investigated factors in the literature [25,26]. In this study, a shoulder finish line configuration was used. A recent study examined the effect of the finish line configuration on the marginal gap adaptation of lithium disilicate crowns and found that the shoulder configuration yielded the lowest marginal gap [27]. Another important factor about finish line configuration is the difference between vertical and horizontal preparations. While some studies have reported that there are no differences between the two finish lines regarding the marginal gap [28,29,30], others have reported that the vertical finish line is less accurate compared to horizontal ones (shoulder type) regarding marginal adaptation [27,31]. 

Yüksel and Zaimoğlu evaluated the effect of different luting cements (RelyX U100 and KETAC CEM) on the marginal gap of several materials (monolithic zirconia, heat-pressed lithium disilicate, and Cr-Co frameworks) and received a mean marginal gap of 92.6 ± 9.53 µm for the lithium disilicate group when cemented with RelyX U100. In the current study, RelyX U200 and ZLS were used, and a marginal gap of 73.83 μm was received. The preparations in both studies were similar, with 6 degrees of convergence and a shoulder finish line of 1.2 mm. The difference can be explained by the fact that the cement was not the same as when we used RelyX U200 compared to RelyX U100. Another difference is that we used digital impressions with Primescan compared to conventional impression with polyether impressions [8].

Several studies have investigated whether digital or conventional impressions may affect the marginal gap. While some studies have found no differences regarding the marginal gap [32,33], others did find differences between the two techniques [34]. 

These research results show that there was a significant difference between the G-Cem ONE group and the TheraCem and RelyX U200 groups regarding the marginal gap. This can be explained by the different filler geometry and viscosity between the three self-adhesive resin cements. Soliman et al. used CELTRA^®^ DUO crowns that were cemented with low-viscosity composites and high-viscosity composites and evaluated the marginal gap width (MGW) using SEM. They found that the MGW was significantly lower for the low-viscosity group [35], and it is known that the viscosity is also affected by the filler content and geometry [21]. In the current literature, we were not able to find studies that investigated the filler content and geometry of these three self-adhesive resin cements. Therefore, it would be very important and interesting to explore this field in future studies.

The mechanism observed in self-adhesive resin cement involves a dynamic balance between acidic hydrophilic monomers, which are responsible for primary etching, and conventional hydrophobic monomers, which are responsible for long-term stability. The PH of the cements affects the polymerization mechanism, which may lead to changes in the viscosity of the cement; hence, it affects the marginal gap [36].

The viscosity of cement depends on the formulation, and it is known that the viscosity of cement can affect the fatigue performance of glass ceramic crowns [37,38]. 

In this research, we used a horizontal preparation, but, in the clinic, we also used vertical preparations, whereby, in some cases, the finish line is on the cementum and not in the dentin. In such cases, an increased depth of cure is needed, and we always want the lowest polymerization shrinkage, which, today, can reach 3–7%. This may increase the marginal gap. Therefore, when a marginal seal needs to be achieved in the cementum, the use of a bulk fill resin-based composite might be considered [39,40].

Materials such as self-adhesive resin cement have gained popularity among clinicians due to their ease of use. The chemical and physical properties of self-adhesive resin cement are like those of non-self-adhesive resin cement and non-resin-based dental cement [41]. Nevertheless, there is a lack in the literature regarding the long-term influences on the marginal gap.

Due to the results of this study, future works could examine other types of cements, different finish line configurations (vertical versus horizontal), other types of IOS, and other types of measurements and materials. 

Several limitations of this study must be mentioned: We used only one type of measurement (SEM); we used only one type of intra oral scanner; it was an in vitro design; we used one type of material and plastic teeth, one type of finish line configuration, and only twelve measurements for each unit.

## 5. Conclusions

With the limitations of this study, the following conclusions can be drawn:

1. All four groups yielded a marginal gap that is within the clinically accepted values, which are less than 120 μm.
2. All three self-adhesive resin cements caused significant increases in the marginal gap compared to the control group.
3. The lowest mean marginal gap was obtained at the temp-bond control group (Temp-Bond™ NE™ Unidose; KaVo Kerr, Brea, CA, USA), and the highest mean marginal gap was obtained at the RelyX U200 Automix group (RXU200; 3M ESPE, Seefeld, Germany).
4. Regarding the marginal gap, all three self-adhesive resin cements can be used, but the most recommended one for CELTRA^®^ DUO single crown regarding the marginal gap is G-cem ONE Automix (GCA; GC, Alsip, IL, USA).

## Figures and Tables

**Table 1:** Median, P25 (25 percentile), P50 (50 percentile), and P75 (75 percentile), minimum, and maximum of the distal, mesial, palatal, and buccal mean marginal gap (μm) of the four groups: control (temp-bond); G-cem ONE; TheraCem; and RelyX U200.

	Distal Surface			Mesial Surface			Palatal Surface			Buccal Surface		
Mean Marginal Gap (MMG)	Median	P25 P50 P75	Min Max	Median	P25 P50 P75	Min Max	Median	P25 P50 P75	Min Max	Median	P25 P50 P75	Min Max
Control Temp-Bond (μm)	25.26	19.26 25.26 29.40	14.29 35.87	25.86	20.41 25.86 28.72	15.61 44.55	25.47	22.19 25.47 28.72	17.64 33.15	21.93	17.47 21.93 27.14	12.16 43.00
G-cem ONE (μm)	46.95	41.98 46.95 52.93	29.64 71.07	50.85	47.28 50.85 60.00	27.50 64.68	49.20	45.78 49.20 55.53	35.48 84.44	49.20	37.33 49.20 60.01	31.02 79.82
TheraCem (μm)	65.87	54.84 65.87 70.56	47.30 90.57	70.44	63.35 70.44 78.83	42.52 84.41	68.94	57.74 68.94 72.23	45.69 88.34	59.24	55.53 59.24 72.54	43.55 92.56
RelyX U200 (μm)	71.57	64.11 71.57 80.73	42.96 100.94	77.79	63.32 77.79 85.88	46.25 90.02	70.48	67.49 70.48 76.53	38.69 84.18	70.34	58.32 70.34 75.23	36.09 87.18

**Table 2:** Median, P25 (25 percentile), P50 (50 percentile), and P75 (75 percentile), minimum, and maximum of the total mean marginal gap (μm) of the four groups: control (temp-bond); G-cem ONE; TheraCem; and RelyX U200.

Total Mean Marginal Gap (TMMG)	Median	P25 P50 P75	Min Max
Control Temp-Bond (μm)	23.60	22.57 23.60 26.12	16.86 35.29
G-cem ONE (μm)	50.66	43.99 50.66 56.89	37.67 58.36
TheraCem (μm)	66.87	59.09 66.87 69.54	54.49 84.47
RelyX U200 (μm)	73.83	63.55 73.83 77.09	42.76 80.69

## Data Availability

All data generated or analyzed during this study are included in this published article.
